# Indigo Carmine and 2,6-Dichlorophenolindophenol Removal Using Cetyltrimethylammonium Bromide-Modified Palm Oil Fiber: Adsorption Isotherms and Mass Transfer Kinetics

**DOI:** 10.1155/2019/6862825

**Published:** 2019-12-13

**Authors:** Marcel Cédric Deussi Ngaha, Evangéline Njanja, Giscard Doungmo, Arnaud Tamo Kamdem, Ignas Kenfack Tonle

**Affiliations:** Electrochemistry and Chemistry of Materials, Department of Chemistry, Faculty of Science, University of Dschang, P.O. Box 67, Dschang, Cameroon

## Abstract

In the present work, the usefulness of cetyltrimethylammonium bromide-modified palm oil fiber (CTAB-modified POF) for the removal of indigo carmine (IC) and 2,6-dichlorophenolindophenol (2,6-DCPIP) from aqueous solutions was investigated. Raw, NaOH-treated, and CTAB-modified POF were characterized by Fourier-transform infrared (FT-IR) spectroscopy, elemental analysis, thermogravimetric-hyperdifferential scanning calorimetric (TG-HDSC) analysis, X-ray diffraction (XRD), and scanning electron microscopy (SEM). The adsorption studies of IC and 2,6-DCPIP were performed in batch mode using CTAB-modified POF. The results showed that equilibrium was attained after a contact time of 30 minutes for IC and 20 minutes for 2,6-DCPIP. The maximum capacity of adsorption was obtained at pH = 2. The capacity of adsorption considerably increased with modified biosorbents and with increasing initial concentration of dyes. The ionic strength favors the increasing adsorption capacity of IC and does not affect the adsorption capacity of 2,6-DCPIP. The percentage of adsorption increased with increasing mass of the biosorbents. The nonlinear regression of adsorption isotherms showed that Freundlich (*r*^2^ = 0.953; *χ*^2^ = 4.398) and Temkin (*r*^2^ = 0.986; *χ*^2^ = 1.196) isotherms are most appropriate to describe the adsorption of IC and 2,6-DCPIP on CTAB-modified POF, respectively. The maximum adsorption capacities determined by the Langmuir isotherm were 275.426 and 230.423 *μ*mol·g^−1^ for IC and 2,6-DCPIP, respectively. The linear regression of adsorption kinetics was best described by the pseudo-second-order model (*R*^2^ ≥ 0.998). The diffusion mechanism showed that external mass transfer is the main rate controlling step. Desorption of the two dyes is favorable in the alkaline medium.

## 1. Introduction

Most dyes are organic compounds having complex aromatic structures with a variety of functional groups and are extensively used in various industries like textile, paper, plastic, food, printing, cosmetic, and pharmaceutical industries [1–3]. However, some dyes discharged from the effluents of the industries into receiving streams are sources of water pollution because most of the dyes and their degradation products are highly toxic, mutagenic, carcinogenic, and allergenic [4–7]. The dyes in water effluents have a very hazardous impact on the environment, making it unfit for human and aquatic life, thus causing chronic and acute diseases [3–8]. Therefore, several treatment methods have been adopted for removal of dyes from wastewaters in view to prevent continuous environmental pollution.

The traditional methods used for removing dyes from wastewaters include flocculation, electroflotation, chemical precipitation, electrokinetic coagulation, ion exchange, membrane filtration, electrochemical destruction, irradiation, ozonation, and microbial biodegradation [9, 10]. However, application of these methods is restricted because they are ineffective and noneconomical and have many disadvantages such as high reagent and energy requirements, generation of toxic sludge, or other waste products that require disposal or treatment [6, 11]. The adsorption technique is the most used in industries for advanced wastewater treatment because it is efficient, simple to design, and does not produce any sludge [1, 12, 13]. The commercial activated carbon has been the first adsorbent used in adsorption. Because of high cost of activated carbon and problems of regeneration of adsorbents after adsorption [14], several research studies were conducted to the use of new adsorbents that are lower in cost, locally available, and efficient [3, 12]: they are lignocellulosic materials. Ever since, adsorption of some dyes on many lignocellulosic materials such as rice husk, orange peel, coconut husk, barley husks, peanut hull, eggshell, cocoa shell [13], hazelnut shells [15], olive stone [16], sugarcane bagasse [17], palm tree trunk [3], and wood sawdust [18] have been investigated, and all of these are effective for dyes removal. However, the modification of these lignocellulosic materials also seems necessary to improve their performance. Some earlier works mentioned that the modification of biosorbents with cationic surfactants is an effective technology to enhance the adsorption capacity of anionic dyes [7, 19–22].

The aim of our work is to valorize the palm grove residues for the biosorption of some organic dyes. In our previous work, we investigate about a natural- and acetic acid-treated palm tree trunk for the adsorption of 2,6-DCPIP [3]. The present study aims to evaluate the potentialities of palm oil fiber modified with cationic surfactants, such as cetyltrimethylammonium bromide, in order to enhance the adsorption capacity towards IC and 2,6-DCPIP from aqueous solutions. The effects of some important parameters such as modification of biosorbents, initial dye concentration, pH of solutions, adsorbent dosage, ionic strength, and contact time on the adsorption of both IC and 2,6-DCPIP onto CTAB-modified POF were compared. The adsorption was described by using adsorption isotherms and kinetic models. The possibility of dyes recovering and to reuse the biosorbent was also investigated.

## 2. Materials and Methods

### 2.1. Materials, Preparations, and Characterizations of Biomass

All chemical reagents used in this experiment were obtained commercially and used without further purification. NaCl and NaOH were purchased from Fisher, IC dye content 85% was purchased from Sigma-Aldrich, HCl was purchased from Phillip Harris, AgNO_3_ was purchased from Kem Light, HNO_3_ and 2,6-DCPIP were purchased from Riedel-de-Häen, and CTAB was purchased from Prolabo.

The palm oil fiber used in this work was kindly provided by a local palm oil producer in the Littoral region of Cameroon. The material was cleaned, cut into small pieces, washed several times with tap water to remove impurities such as sand, dust, and soil particles, and then dried for 8 days in sunlight. The dried biomass was ground into fine powder and sieved to obtain sizes lower than 100 *μ*m for further analyses.

Prior to the modification with CTAB, the raw POF was first submitted to pretreatment with NaOH solution, according to the following procedure: 5 g of raw POF was stirred on a mechanical platform shaker (Edmund Bühler GmbH) in 100 mL of 2 mol·L^−1^ NaOH solution for 2 h at a speed of 200 rpm. The obtained material was washed several times with distilled water (in order to remove the excess of NaOH until the neutral pH of washed water was measured), air-dried for 2 days, and then placed in an oven at 110°C for 24 h before use.

For the modification with CTAB, 1 g of NaOH-treated POF freshly removed from the oven was dispersed in 100 mL of 14 mmol·L^−1^ CTAB solution. The whole was also agitated on a mechanical platform shaker (Edmund Bühler GmbH) for a time period of 24 h at a speed of 200 rpm. Afterwards, the suspension was filtered, and the resultant material was washed several times with distilled water for the removal of superficially retained CTAB until no white precipitate was formed on addition of AgNO_3_ (0.1 mol·L^−1^) to the washed water. The obtained biosorbent was dried in an oven at 60°C for 24 h before being sieved and kept in a bottle for further use.

The raw, NaOH-treated, and CTAB-modified POF were characterized using Fourier-transform infrared spectroscopy, elemental analysis, thermogravimetric-hyperdifferential scanning calorimetric analysis, X-ray diffraction analysis, and scanning electron microscopy. The FT-IR spectra were obtained by means of the ATR technique with a FT-IR spectrophotometer (Bruker *α*-P, Germany) within a range of 4000–400 cm^−1^ and with a resolution of 4 cm^−1^; 200 scans were collected for each spectrum. The ultimate analyses of elemental analysis were performed with an Elemental Analyzer (HEKAtech CHNS, Germany). The results were obtained as percentages of carbon, hydrogen, and nitrogen, and the oxygen content was determined indirectly by difference to 100%. Thermogravimetric-hyperdifferential scanning calorimetric (TG-HDSC) analyses were performed using a thermal analyzer (LINSEIS STA PT-1000, Germany) at a heat rate of 10°C·min^−1^ starting from room temperature to a maximum temperature of 700°C under air atmosphere. X-ray diffraction analyses were carried out using a STOE Stadi-p X-ray powder diffractometer (STOE & Cie GmbH, Darmstadt, Germany) with CuKα radiation (40 kV, 30 mA and  *λ*=0.54056 Å). XRD patterns were recorded between 0° and 70° 2*θ*°, ranging in steps of 0.033° with a counting time per step of 200 s and with a scanning rate of 5° min^−1^. SEM pictures were taken using a scanning electron microscope (Amray 1610 Turbo, USA).

### 2.2. Batch Adsorption and Desorption Studies

The stock solutions were prepared at a concentration of 1 × 10^−3^ mol·L^−1^ by dissolving an accurately weighed amount of 0.2332 g of sodium salt of indigo carmine or of 0.1631 g of the hydrated sodium salt of 2,6-dichlorophenolindophenol in 500 mL of distilled water. The experimental solutions (1 × 10^−5^–1 × 10^−4^ mol·L^−1^) were prepared by diluting the stock solutions with distilled water. The pH of each dye solutions was adjusted to the required value along the experiment by using small aliquots of HCl or NaOH solutions.

In this study, all adsorption studies were achieved in aqueous solution and at room temperature of 24.5°C. Batch adsorption experiments were implemented in a set of well-adapted flasks in which various preweighted amounts (1–14 mg) of biosorbent were contacted with 30 mL of dye solutions of various initial concentrations (1 × 10^−5^– 1 × 10^−4^ mol·L^−1^). The mixture was stirred on a mechanical platform shaker at a constant agitation speed of 150 rpm for a variable specific period of contact time between 2 and 60 min. Afterwards, the supernatant solutions obtained by filtrating the suspensions using the Whatman filter paper were analyzed by measuring the absorbance using UV-Vis Spectrophotometer (JENWAY) at a *λ*_max_ of 610 nm for IC [23] and 600 nm for 2,6-DCPIP [3, 5]. The monitoring absorbance was converted to the corresponding final concentration by using linear regression equations of calibration curve obtained from the linear plots of absorbance versus initial dye concentration of standard solutions. The dye uptake at equilibrium *q*_e_ (mol·g^−1^) and at time *q*_t_ (mol·g^−1^), and the dye removal percentage (%_ads_) was estimated using the following equations [3, 16, 24, 25]:

(1) $$\begin{array}{c} q_{\text{e}}=\frac{\left(C_{\text{i}}-\;C_{\text{e}}\right)}{m}V_{\text{s}},\\ q_{\text{t}}=\frac{\left(C_{\text{i}}-\;C_{\text{t}}\right)}{m}V_{\text{s}},\\ {\%}_{\text{ads}}=\frac{\left(C_{\text{i}}-\;C_{\text{e}}\right)}{C_{\text{i}}}\;\times \;100, \end{array}$$

where *C*_i_ (mol·L^−1^) is the initial dye concentration, *C*_e_ (mol·L^−1^) is the equilibrium final dye concentration, *C*_t_ is the final dye concentration at time *t*, *V*_s_ (L) is the dye solution volume, and *m* (g) is the biosorbent weight.

For the desorption studies, 20 mg of CTAB-modified POF was combined in a flask with 100 mL of each dye solution at an initial concentration of 5 × 10^−5^ mol·L^−1^. After stirring the mixtures at a constant agitation speed of 150 rpm for a period of 40 min for IC and 30 min for 2,6-DCPIP, the suspensions were filtered using the Whatman filter paper and the supernatant solutions obtained were analyzed by measuring the absorbance using UV-Vis Spectrophotometer. The dyes loaded with CTAB-modified POF, were collected after filtration, and were dried in an oven at 60°C, and 4 mg of each of these materials was contacted with 30 mL of desorption solutions of H_2_O and 1 × 10^−4^ mol·L^−1^ of NaOH or HNO_3_. Afterwards, the mixtures were also stirred in the same condition as described earlier and the suspensions were again filtered. The supernatant solutions were also analyzed as described earlier in view to determine the desorbed dye concentration. The desorption percentages (%_des_), translating the fraction of dyes released by the material, were determined as follows [3, 26]:

(2) $$\begin{array}{c} {\%}_{\text{des}}=\frac{C_{\text{f}}-C_{\text{r}}}{C_{\text{f}}}\times 100, \end{array}$$

where *C*_f_ (mol·L^−1^) is the initial concentration of dyes loaded with CTAB-modified POF and *C*_r_ (mol·L^−1^) is the final concentration of dyes loaded with CTAB-modified POF.

### 2.3. Error Analysis

The nonlinear regression coefficient of determination (*r*^2^) and chi-square test (*χ*^2^) were used to evaluate the applicability of adsorption isotherm models. The expressions of the error functions are given as follows [27–30]:

(3) $$\begin{array}{c} \chi^{2}=\overset{N}{\underset{1}{\sum }}\frac{{\left(q_{\text{e,exp}}-\;q_{\text{e,cal}}\right)}^{2}}{q_{\text{e,cal}}},\\ r^{2}=\;\frac{\sum_{1}^{N}{\left(q_{\text{e,cal}}-\;\bar{q_{\text{e,exp}}}\right)}^{2}}{\sum_{1}^{N}{\left(q_{\text{e,cal}}-\;\bar{q_{\text{e,exp}}}\right)}^{2}+\sum_{1}^{N}{\left(q_{\text{e,cal}}-\;q_{\text{e,exp}}\right)}^{2}}, \end{array}$$

where *q*_e,exp_  and *q*_e,cal_ (*μ*mol·g^−1^) are the equilibrium capacity of adsorption obtained from the experiment and by calculating from the model, respectively. *N* is the number of observations in the regression model (the number of data points).

## 3. Results and Discussion

### 3.1. Characterization of Biosorbent

#### 3.1.1. Fourier-Transform Infrared (FT-IR) Spectroscopy

**Figure 1:**
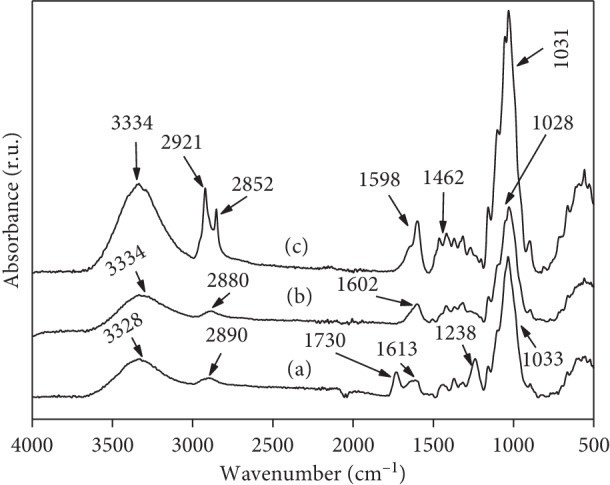
FT-IR spectra of (a) raw POF, (b) NaOH-treated POF, and (c) CTAB-modified POF.

The FT-IR spectrum examination of raw POF was undertaken in order to locate the surface chemical functional groups, as shown in Figure 1(a). The spectrum exhibits the presence of a broad peak in the region 3400–3200 cm^−1^, characteristics of the OH group, indicating the presence of alcohol, phenol, or carboxylic acids [3, 31, 32]. The band at 2890 cm^−1^ was assigned to the presence of asymmetric and symmetric vibrations of CH_2_. The carbonyl (C=O) of carboxylic acids and carboxylate groups occurred, respectively, at 1730 cm^−1^ and 1613 cm^−1^. Another absorption peak at 1238 cm^−1^ could be attributed to C-O, C-H, or C-C stretching vibrations of carboxyl acid groups (-COOH). The maximum adsorption band localized around 1033 cm^−1^ is probably due to C-O stretching vibrations of lignin [3, 31, 32].

The FT-IR spectrum of NaOH-treated POF (Figure 1(b)) reveals that the peaks at 1730 cm^−1^ and 1238 cm^−1^ located in raw POF disappeared after NaOH treatment. Moreover, the peak observed at 1613 cm^−1^ changed from lower intensity peak in the IR spectrum of raw POF to higher intensity peak in the IR spectrum of NaOH-treated POF [32–34].

Compared to the FT-IR spectrum of NaOH-treated POF (Figure 1(b)), the FT-IR spectrum of CTAB-modified POF (Figure 1(c)) reveals the presence of new peaks at wavelength bands of 2921 cm^−1^ and 2852 cm^−1^. These bands are associated with the symmetric and asymmetric stretching vibrations of the methylene (CH_2_) and methyl group (CH_3_) of the aliphatic chain of the surfactant. Furthermore, another new peak of weak intensity appeared at 1462 cm^−1^, which was attributed to methyl groups of the cationic substituent [7, 20, 21].

#### 3.1.2. Thermogravimetric-Hyperdifferential Scanning Calorimetry (TG-HDSC)

**Figure 2:**
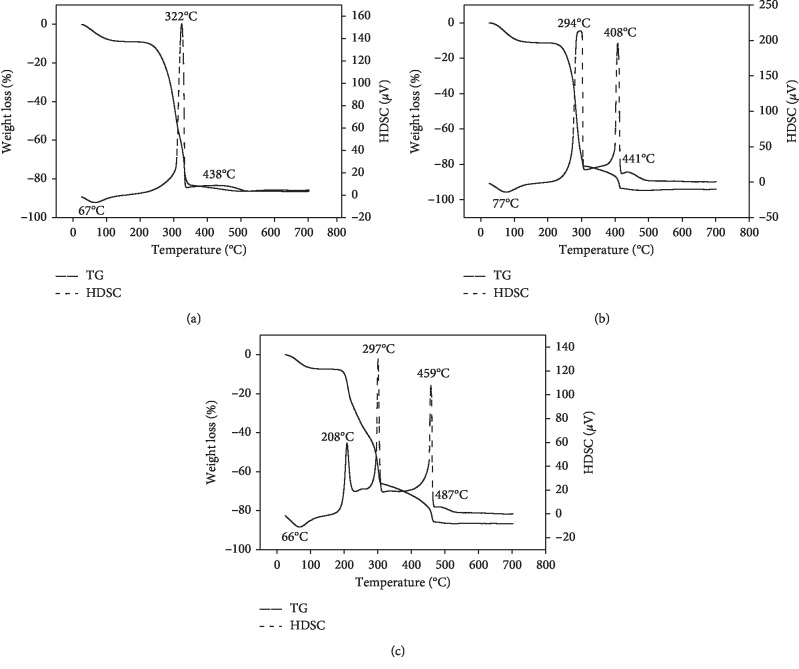
TG-HDSC curves of (a) raw POF, (b) NaOH-treated POF, and (c) CTAB-modified POF.

The thermal analyses of raw, NaOH-treated, and CTAB-modified POF were performed in terms of TG-HDSC curves. As shown in Figure 2(a), the TG-HDSC curves of raw POF suggest four typical distinct weight losses. The first weight loss of 8.92% located around 67°C over a temperature range of 26°C–200°C was attributed to the dehydration or loss of physically adsorbed water and water molecules. The second weight loss of 42.89% within the temperature range of 200°C–300°C may be due to the predominant decomposition of hemicelluloses, followed by a third high weight loss of 30.64% in the range of 300°C–350°C which was attributed to decomposition of cellulose. The last small weight loss of 2.52% above 350°C and located around 438°C was mostly due to decomposition of lignin [35–38].

The TG-HDSC curves of NaOH-treated POF (Figure 2(b)) also exhibited four typical distinct weight losses, which correspond to the thermal decomposition of the same elements in raw POF. The first weight loss of 10.89%, which occurred over a temperature interval of 26°C–200°C, was due to adsorbed water. The second weight loss of 69.51% over a temperature range of 200°C–310°C was due to the elimination of hemicellulose. The third weight loss of 12.49% within a temperature range of 310°C–420°C was due to the thermal alteration of cellulose. The last weight loss of 1.01% above 420°C was due to the thermal degradation of lignin.

In order to verify the effective modification of NaOH-treated POF by CTAB, the thermal analysis characterization of CTAB-modified POF was also performed and the results showed that the TG-HDSC curves of CTAB-modified POF (Figure 2(c)) exhibited five typical distinct weight losses. The first weight loss of 7.36%, which occurred over a temperature interval of 26°C–200°C was due to adsorbed water. The decrease in the first weight loss of CTAB-modified POF (7.36%) compared to NaOH-treated POF (10.89%) is due to the hydrophobic nature of POF after insertion of the CTAB in the interlayer of this biosorbent. The second weight loss of 20.41% over a temperature range of 200°C–250°C was due to the elimination of hemicellulose. The third weight loss of 27.10% over a temperature interval of 250°C–330°C which was absent on the curves of NaOH-treated POF was related to the evaporation/decomposition of the loaded surfactants (CTAB; whose melting point is between 248°C and 251°C) in the interlayer of biosorbent [19, 22]. The fourth weight loss of 13.39% within a temperature range of 250°C–300°C was due to the thermal alteration of cellulose. The last weight loss above 480°C that is nonsignificant was due to the thermal degradation of lignin.

#### 3.1.3. Scanning Electron Microscopy (SEM)

**Figure 3:**
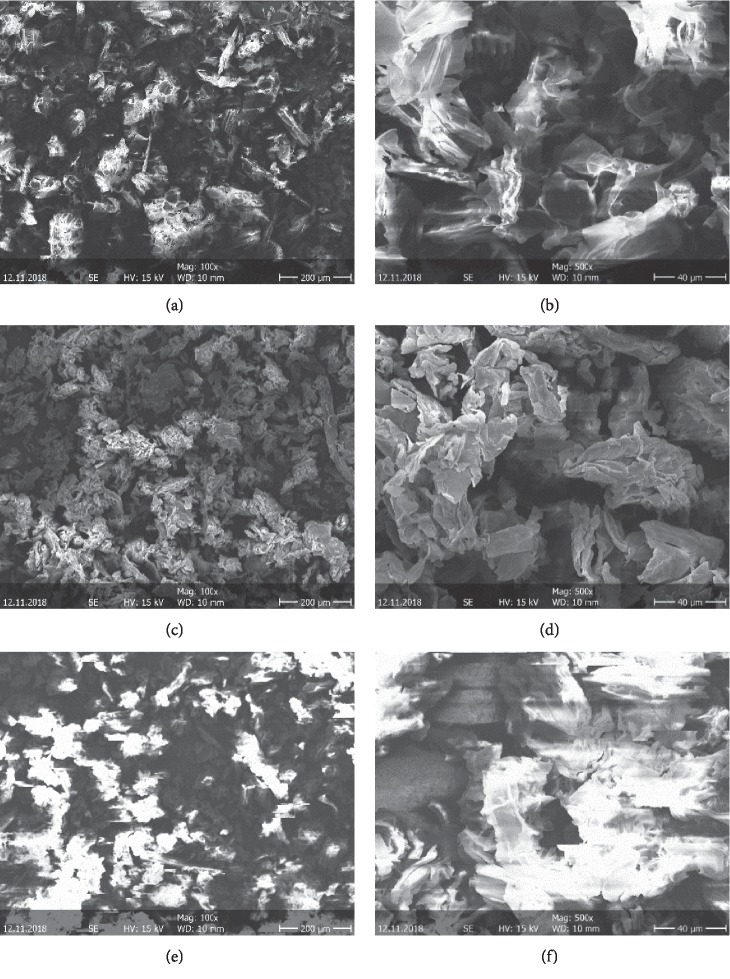
SEM images of (a, b) raw POF, (c, d) NaOH-treated POF, and (e, f) CTAB-modified POF.

Scanning electron microscopy of raw, NaOH-treated, and CTAB-modified POF was carried out in view to assess the effect of NaOH treatment and CTAB modification on the surface morphology of POF. As shown in Figures 3(a) and 3(b), the surface morphology of raw POF presents an irregular shape and a porous surface. After NaOH treatment (Figures 3(c) and 3(d)), the biosorbent (NaOH-treated POF) has a spongy morphology with a porosity more developed due to the action of NaOH on POF that promotes highest weight losses of biosorbent which leads to an increase in internal surface area and also favors the development of pores [39, 40]. The SEM image of CTAB-modified POF (Figures 3(e) and 3(f)) exhibited a surface almost smooth, more compact, or again that contains very few pores due to the fact that the external surface of CTAB-modified POF was covered by a CTAB thin film [7].

#### 3.1.4. X-Ray Diffraction Analysis (XRD)

**Figure 4:**
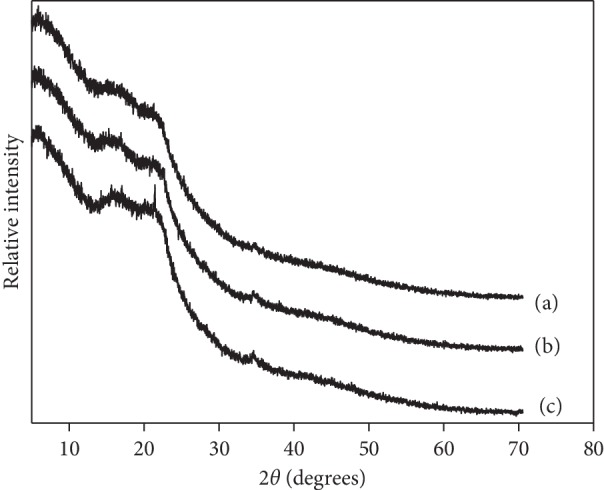
XRD curves of (a) raw POF, (b) NaOH-treated POF, and (c) CTAB-modified POF.

X-ray diffraction technique is a powerful tool used to investigate the crystalline nature of materials. As shown in Figure 4(a), in the X-ray diffraction pattern of raw POF, the peaks are observed at the 16°, 22.5°, and 34.5° 2*θ* angles, corresponding to the cellulose I structure. These peaks are indicative of highly organized crystalline cellulose [41, 42]. The last-mentioned peaks are also observed in the XRD pattern of NaOH-treated (Figure 4(b)) and CTAB-modified POF (Figure 4(c)), indicating that the crystal structure remains stable after NaOH treatment and CTAB modification processes.

#### 3.1.5. Elemental Analysis

The elemental analyses of raw, NaOH-treated, and CTAB-modified POF are illustrated in Table 1. As shown in Table 1, the raw POF is composed of 42.282% carbon, 6.276% hydrogen, 0.788% nitrogen, and 50.654% oxygen. After the NaOH treatment, the biosorbent is composed of the same chemical elements: 40.532% carbon, 6.161% hydrogen, 0.263% nitrogen, and 53.044% oxygen. It can be observed that the percentages of carbon, hydrogen, and nitrogen of NaOH-treated POF are lower than those of raw POF, while the oxygen percentage of NaOH-treated POF is higher than that of raw POF. A similar trend was also obtained for the NaOH treatment of olive tree pruning by Calero et al. [39] and Ronda et al. [43]. Likewise, after the CTAB modification, the biosorbent is also composed of the same chemical elements: 49.236% carbon, 8.006% hydrogen, 1.229% nitrogen, and 41.529% oxygen. It can also be observed that the percentages of carbon, hydrogen, and nitrogen for CTAB-modified POF are higher than those of NaOH-treated POF, while the oxygen percentage of CTAB-modified POF is lower than that of NaOH-treated POF. This increase in carbon, hydrogen, and nitrogen percentages is expected due to the loading of CTAB in the NaOH-treated POF interlayer [44].

### 3.2. Effect of Modification of Biosorbent

**Figure 5:**
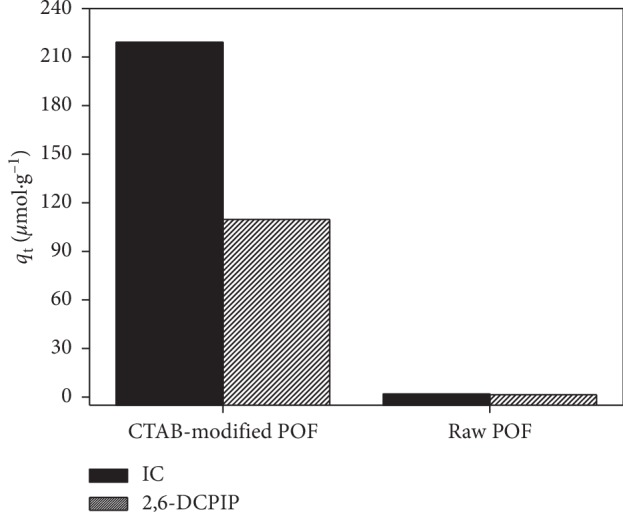
Effect of CTAB-modified POF on the IC and 2,6-DCPIP adsorption. Experimental conditions: [IC] = [2,6-DCPIP] = 5 × 10^−5^ mol·L^−1^; *m* = 0.133 g·L^−1^; *G* = 0–100 *μ*m; *t* = 60 min; *v* = 150 rpm; V = 30 mL; pH = 6.2 for IC and pH = 6.7 for 2,6-DCPIP; at room temperature.

The biosorbent was first of all treated with NaOH to be modified by CTAB.  Figure 5 shows the effect of chemical modification on the adsorption of IC and 2,6-DCPIP. The amount of dyes adsorbed at equilibrium is higher with CTAB-modified POF (219.07 and 109.686 *μ*mol·g^−1^ for IC and 2,6-DCPIP, respectively) than that obtained with raw POF (1.898 and 1.450 *μ*mol·g^−1^ for IC and 2,6-DCPIP, respectively). This result can be explained by the fact that the NaOH treatment promotes high weight losses and hydrolysis reactions that cause high dissolution of organic substances from the biomass. These hydrolysis reactions lead to the formation of more carboxylate (-COO^−^) groups in the NaOH-treated biomass [32, 33, 39], which enhance the cationic surfactant fixation by electrostatic attraction. In addition, NaOH treatment of raw POF causes the swelling of material which leads to an increase in the internal surface area and also favors the cationic surfactant fixation [12]. The increase in cationic surfactant fixation after NaOH treatment of raw POF could also be due to the destruction of autolytic enzymes causing putrefaction of biomass and the removal of lipids and proteins as well as polysaccharides that mask binding sites [32, 45]. The results of the adsorption of IC and 2,6-DCPIP onto CTAB-modified NaOH-treated POF and CTAB-modified raw POF (figure not shown here) were 219.070 and 140.098 *μ*mol·g^−1^ for IC and 109.686 and 58.028 *μ*mol·g^−1^ for 2,6-DCPIP, respectively. This indicates that CTAB-modified NaOH-treated POF has higher adsorption capacities than CTAB-modified raw POF due to the NaOH treatment.

**Figure 6:**
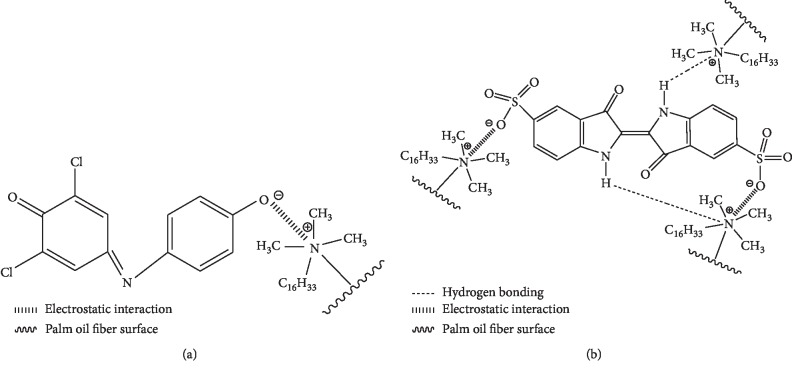
Schematic representation of adsorption mechanisms of (a) 2,6-DCPIP and (b) IC onto CTAB-modified POF.

Secondly, the modification of POF by CTAB covered the surface of cell walls by cationic surfactant which increased new functional groups on the POF surfaces and facilitated the formation of hydrogen bonding and electrostatic interaction between the positively charged surface of the material and the negatively charged molecules of anionic dyes (Figure 6) [7]. In addition, when the CTAB is inserted into the POF interlayer, the POF became hydrophobic, improving its adsorption capacity [19, 21].

### 3.3. Effect of Biosorbent Dosage

**Figure 7:**
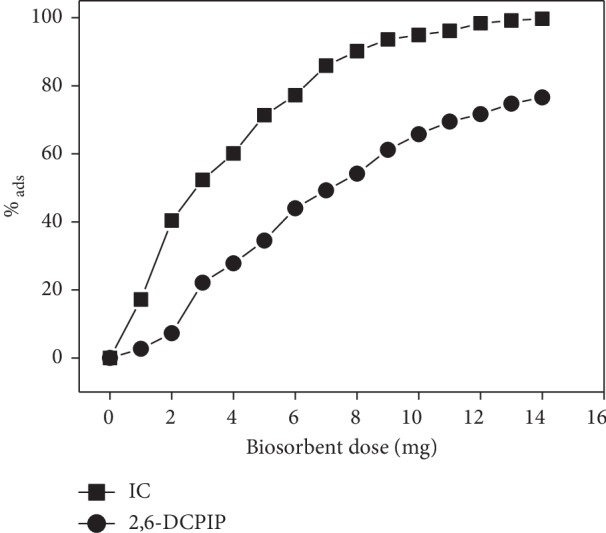
Effect of biosorbents dose on the IC and 2,6-DCPIP adsorption. Experimental conditions: [IC] = [2,6-DCPIP] = 5 × 10^−5^ mol·L^−1^; *G* = 0–100 *μ*m; *t* = 40 min and pH = 6.2 for IC; *t* = 30 min and pH = 6.7 for 2,6-DCPIP; *v* = 150 rpm; *V* = 30 mL; at room temperature.

The effect of biosorbent dosage on the removal of IC and 2,6-DCPIP at an initial dye concentration was studied, and the results are shown in Figure 7. The removal percentage of IC and 2,6-DCPIP increased up to 99.696% and 76.589%, respectively. As can be seen, the percentage of adsorption increased with increasing adsorbent dosage. This can be explained by the fact that the increase in adsorbent dosage involves a greater surface area and more availability of free adsorption sites for dye adsorption during the adsorption reaction [25, 46, 47].

### 3.4. Effect of Contact Time

**Figure 8:**
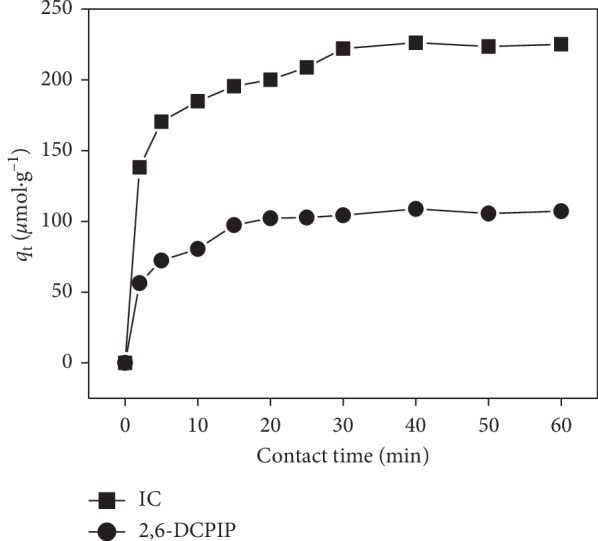
Effect of contact time on the IC and 2,6-DCPIP adsorption. Experimental conditions: [IC] = [2,6-DCPIP] = 5 × 10^−5^ mol·L^−1^; *m* = 0.133 g·L^−1^; *G* = 0–100 *μ*m; *v* = 150 rpm; *V* = 30 mL; pH = 6.2 for IC and pH = 6.7 for 2,6-DCPIP; at room temperature.

The effect of contact time (2–60 min) on adsorption of IC and 2,6-DCPIP is presented in Figure 8. Rapid adsorption of IC and 2,6-DCPIP takes place in the first 2 minutes for the two dyes; thereafter, the adsorption rate gradually reduces with the increasing adsorption time until reaching equilibrium in about 30 minutes for IC (222.107 *μ*mol·g^−1^) and 20 minutes for 2,6-DCPIP (102.306 *μ*mol·g^−1^). Beyond the equilibrium time, we observed no significant change in dye removal. The initial rapid phase may be attributed to rapid dye attachment on the CTAB-modified POF surface due to either the large amount of surface area available or to the availability of more adsorption vacant sites at the initial stage. Subsequently, the lowering of the rate of the adsorption is due to the decrease in the total adsorbent surface area and less available binding sites [1, 17]. No change in the amount of dye adsorbed after the equilibrium period is due to the saturation of the adsorption active sites by the dye molecules [1, 25, 48].

### 3.5. Effect of Initial Dye Concentration

**Figure 9:**
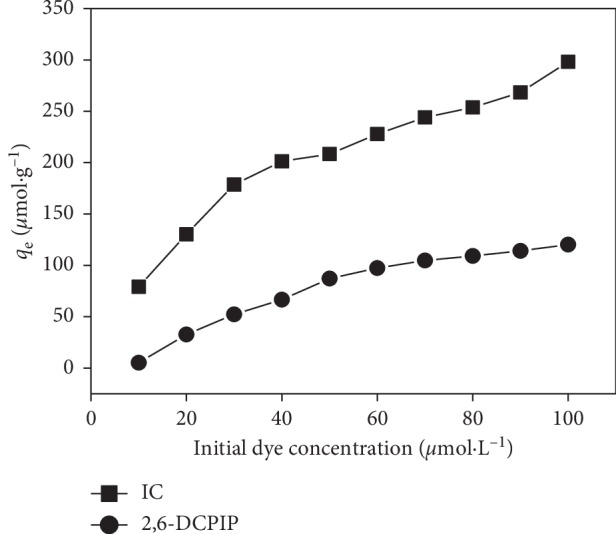
Effect of initial concentration on the IC and 2,6-DCPIP adsorption. Experimental conditions: *m* = 0.133 g·L^−1^; *G* = 0–100 *μ*m; *t* = 40 min and pH = 6.2 for IC; *t* = 30 min and pH = 6.7 for 2,6-DCPIP; *v* = 150 rpm; *V* = 30 mL; at room temperature.

The effect of initial concentration on the adsorption of IC and 2,6-DCPIP was investigated in the range of 10–100 *μ*mol·L^−1^. As shown in Figure 9, the amount of IC and 2,6-DCPIP adsorbed at equilibrium increases from 79.176 to 298.191 *μ*mol·g^−1^ and from 5.249 to 120.209 *μ*mol·g^−1^, respectively. The amount adsorbed at equilibrium increases with the concentration of both dyes. This result can be explained by the fact that increasing the initial dye concentration would increase the mass transfer driving force, i.e., concentration gradient, and also favor the rate at which dye molecules pass from solution to the particle surface [4, 9, 25]. This behaviour suggests that available sites on the biosorbent surface are the limiting factor for the IC and 2,6-DCPIP removal [3, 17].

### 3.6. Effect of Solution pH

**Figure 10:**
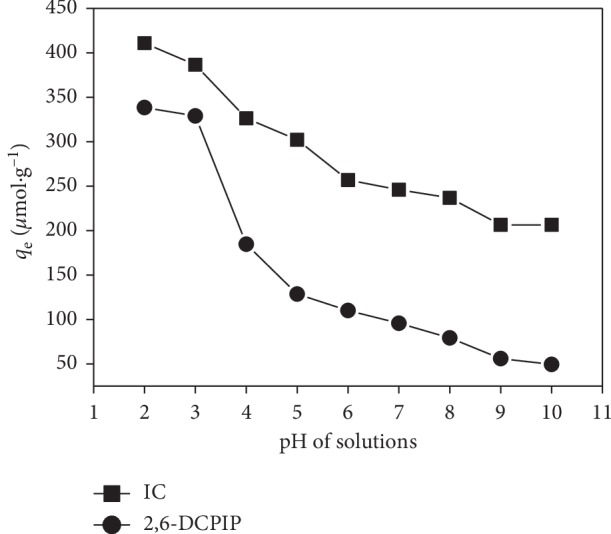
Effect of solution pH on the IC and 2,6-DCPIP adsorption. Experimental conditions: [IC] = [2,6-DCPIP] = 5 × 10^−5^ mol·L^−1^; *m* = 0.133 g·L^−1^; *G* = 0–100 *μ*m; *t* = 40 min for IC; *t* = 30 min for 2,6-DCPIP; *v* = 150 rpm; *V* = 30 mL; at room temperature.

The effect of initial solution pH on adsorption of IC and 2,6-DCPIP onto CTAB-modified POF is shown in Figure 10, and the results indicate that when the pH increases, the capacity of adsorption decreases from 410.804 to 206.426 *μ*mol·g^−1^ and from 338.458 to 49.418 *μ*mol·g^−1^ for IC and 2,6-DCPIP dyes, respectively. This can be explained by the fact that the point of zero charge (PZC, determined by the method described in our previous work by Ngaha et al. [3]) of CTAB-modified POF is 6.6; thus, at low pH (pH < PZC), more protons will be available for the biosorbent surface protonation, which increases the electrostatic attraction between the positively charged biosorbent sites and the negatively charged dyes. At lower pH (pH = 2), there is nearly no electrostatic repulsion between the biosorbent and the dyes; hence, the amount adsorbed is at its maximum. Increasing the pH (pH > PZC) favors the raise in hydroxyl ions, which lead to an increase in dye anions in the solution as well as the number of negatively charged sites on the adsorbent surface. This results in electrostatic repulsion between the dyes and CTAB-modified POF, which is the reason for the decrease in the adsorption capacity [3, 49, 50].

### 3.7. Effect of Ionic Strength

**Figure 11:**
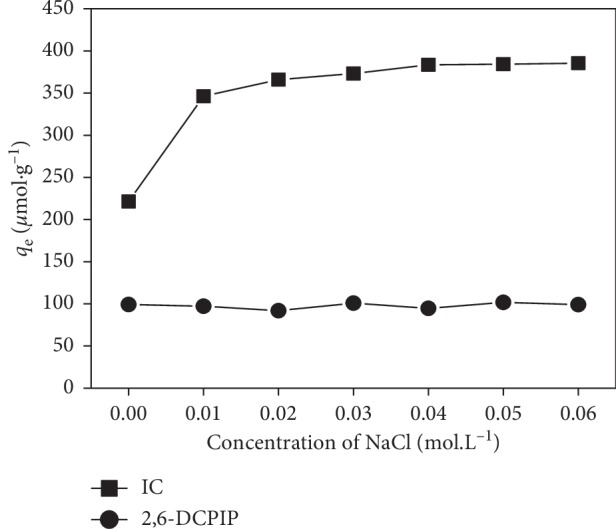
Effect of ionic strength on the IC and 2,6-DCPIP adsorption. Experimental conditions: [IC] = [2,6-DCPIP] = 5 × 10^−5^ mol·L^−1^; *m* = 0.133 g·L^−1^; *G* = 0–100 *μ*m; *t* = 40 min and pH = 6.2 for IC; *t* = 30 min and pH = 6.7 for 2,6-DCPIP; *v* = 150 rpm; *V* = 30 mL; at room temperature.

The effect of ionic strength on adsorption of IC and 2,6-DCPIP onto CTAB-modified POF was analyzed in the NaCl solutions with concentrations ranging from 0.000 to 0.060 mol·L^−1^, and the results are illustrated in Figure 11. When the ionic strength increased, the adsorption capacity of IC increased (from 221.348 to 385.366 *μ*mol·g^−1^) and the adsorption capacity of 2,6-DCPIP is not affected (≈97.784 *μ*mol·g^−1^). The increase in IC adsorption capacity can be attributed to the fact that the addition of salt (NaCl) favors the aggregation of IC molecules and decreases its solubility [3, 51, 52]. Another explanation is that the positive charge surface of biosorbent increases with increasing ionic strength, which leads to an increase in electrostatic attraction between IC ions and CTAB-modified POF [3, 53]. Secondly, the insignificant change in 2,6-DCPIP adsorption capacity may be due to the fact that chlorine and sodium ions have no effect on adsorption of 2,6-DCPIP onto CTAB-modified POF [54].

### 3.8. Adsorption Isotherm Models

#### 3.8.1. Langmuir Isotherm

The general formulas of nonlinear (equation (4)) and linear (equation (5)) expressions of the Langmuir isotherm are, respectively, illustrated as follows [14, 55]:

(4) $$\begin{array}{c} q_{\text{e}}=\frac{K_{\text{L}}q_{\max}C_{\text{e}}}{1+K_{\text{L}}C_{\text{e}}}, \end{array}$$

(5) $$\begin{array}{c} \frac{C_{\text{e}}}{q_{\text{e}}}=\frac{1}{K_{\text{L}}\;.\;q_{\max}}+\frac{C_{\text{e}}}{q_{\max}}, \end{array}$$

where *q*_max_(*µ*mol · g^−1^) is the maximum adsorption capacity of CTAB-modified POF, *q*_e_(*µ*mol · g^−1^) is the equilibrium adsorption capacity of CTAB-modified POF, *C*_e_(*µ*mol · L^−1^) is the concentration of dye solutions at equilibrium, and *K*_L _(L · *µ*mol^−1^) is the Langmuir adsorption equilibrium constant. The linear plots of *C*_e_/*q*_e_ versus *C*_e_ allowed to determine *q*_max_ and *K*_L _ constant values from their slopes and intercepts, respectively. The separation factor (*R*_L_) is defined using the following equation [3, 56]:

(6) $$\begin{array}{c} R_{\text{L}}=\frac{1}{1+K_{\text{L}}C_{\text{i}}}. \end{array}$$

The *R*_L_ value indicates whether the shape of the adsorption isotherm will be either favorable (0 < *R*_L_ < 1), unfavorable ( *R*_L_ > 1), linear (*R*_L_=1), or irreversible (*R*_L_=0).

#### 3.8.2. Freundlich Isotherm

The general formulas of nonlinear (equation (7)) and linear (equation (8)) expressions of the Freundlich isotherm are, respectively, described as follows [14, 55]:

(7) $$\begin{array}{c} q_{\text{e}}=K_{\text{f}}\;C_{\text{e}}^{1/n}, \end{array}$$

(8) $$\begin{array}{c} \text{Log }q_{\text{e}}=\log\;K_{\text{f}}+\frac{1}{n}\log\;C_{\text{e}}, \end{array}$$

where *K*_f _(L · g^−1^) is the Freundlich constant taken as an indicator of adsorption capacity and 1/*n* is a measure related to the adsorption intensity of the CTAB-modified POF. The linear plots of log *q*_e_ versus log *C*_e_ allowed to determine the 1/*n* and *K*_f _ constant values from their slopes and intercepts, respectively.

#### 3.8.3. Dubinin–Radushkevich Isotherm

The general formulas of nonlinear (equation (9)) and linear (equation (10)) equations of the Dubinin–Radushkevich isotherm are, respectively, illustrated by the following expressions [16, 57]:

(9) $$\begin{array}{c} q_{\text{e}}=q_{\max}e^{-\beta \varepsilon^{2}}, \end{array}$$

(10) $$\begin{array}{c} \ln\;q_{\text{e}}=\ln\;q_{\max}\;-\;\beta \varepsilon^{2}, \end{array}$$

where *β* (mol^2^·kJ^−2^) is the activity coefficient related to the mean free energy (*E* (kJ·mol^−1^)) and *ε* is Polanyi potential calculated from the following equation:

(11) $$\begin{array}{c} \varepsilon =\text{RT}\ln\left(1\;+\;\frac{1}{C_{\text{e}}}\right),\\ E=\frac{1}{\sqrt{-2\beta }}, \end{array}$$

where *R* (8.314 × 10^−3^ kJ·mol^−1^·K^−1^) and *T* (298 K) are the universal gas constant and the absolute temperature, respectively. The constant values of *β* and *q*_max_ were evaluated, respectively, from the slopes and intercepts of straight lines obtained by plotting ln *q*_e_ versus *ε*^2^.

#### 3.8.4. Temkin Isotherm

The general formulas of nonlinear (equation (12)) and linear (equation (13)) equations of the Temkin isotherm are, respectively, given by the following expressions [28, 55, 57, 58]:

(12) $$\begin{array}{c} q_{\text{e}}=q_{\max}\;\frac{\text{RT}}{\Delta Q}\ln\;K_{\text{T}}C_{\text{e}}, \end{array}$$

(13) $$\begin{array}{c} q_{\text{e}}=q_{\max}\;\frac{\text{RT}}{\Delta Q}\ln\;K_{\text{T}}+q_{\max}\;\frac{\text{RT}}{\Delta Q}\;\ln\;C_{\text{e}}, \end{array}$$

where Δ*Q*  (kJ·mol^−1^) and *K*_T_(L·*μ*mol^−1^) are the adsorption heat and the Temkin isotherm constant, respectively. The constant values of Δ*Q* and *K*_T_ were determined from the slopes and the intercepts of straight lines obtained by plotting *q*_e_ versus ln *C*_e_, respectively.

**Figure 12:**
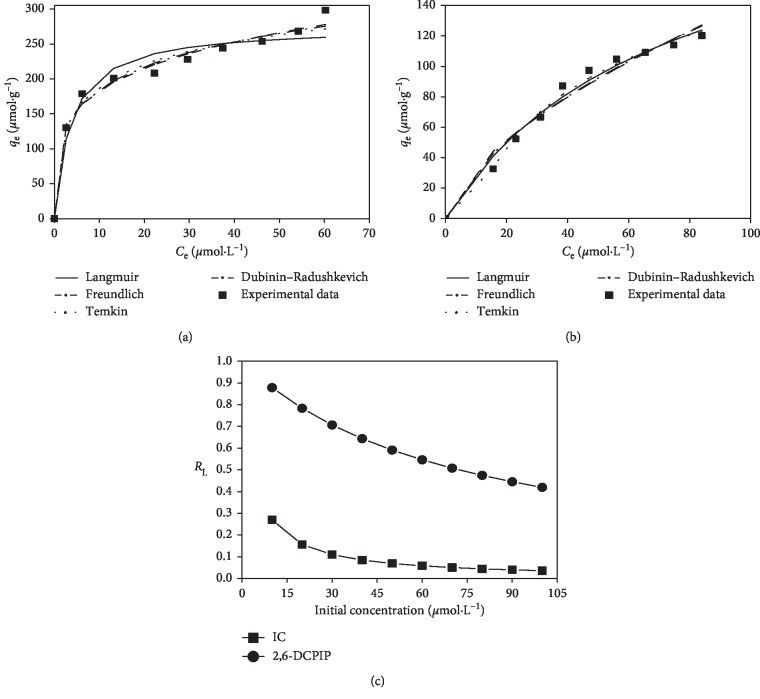
Variation in the adsorbed amount of (a) IC and (b) 2,6-DCPIP at equilibrium on CTAB-modified POF and (c) evolution of *R*_L_ values against the initial dye concentration.

The nonlinear regression and separation factor graphs of adsorption isotherms obtained for IC and 2,6-DCPIP adsorption onto CTAB-modified POF are shown in Figure 12. The corresponding constants obtained from the nonlinear regressions are recapped in Table 2.

The data of Table 2 indicate that the values of coefficient of determination of the Freundlich isotherm (*r*^2^ = 0.953) for IC and Temkin isotherm (*r*^2^ = 0.986) for 2,6-DCPIP are closest to unity, implying that Freundlich and Temkin isotherms are most appropriate to describe the adsorption of IC and 2,6-DCPIP on CTAB-modified POF, respectively. This is also confirmed by the low values of chi-square test error obtained with the Freundlich isotherm (*χ*^2^ = 4.398) for IC and Temkin isotherm (*χ*^2^ = 1.196) for 2,6-DCPIP. The good fit of IC experimental equilibrium data to the Freundlich isotherm indicates the multilayer coverage with heterogeneous sorption sites and different fixing energies of IC onto CTAB-modified POF [16, 58].

It is difficult to compare the maximum adsorption capacity values determined by the Langmuir model of different types of adsorbents because the experimental conditions used are not identical.  Table 3 summarizes the comparison of the maximum IC and 2,6-DCPIP adsorption capacities of various adsorbents including CTAB-modified POF. The comparison shows that CTAB-modified POF has higher adsorption capacity of IC (128.444 mg·g^−1^) and 2,6-DCPIP (75.143 mg·g^−1^) than many of the other reported adsorbents, reflecting a promising future for CTAB-modified POF utilization in IC and 2,6-DCPIP removal from aqueous solutions.

The values of 1/*n* evaluated from the Freundlich model (0.231 for IC and 0.629 for 2,6-DCPIP) are less than unity, meaning that adsorption is favorable for both dyes [3, 14, 55]. The adsorption energy obtained from the Dubinin–Radushkevich isotherm (9.880 kJ·mol^−1^) lies in the range of 8 to 16 kJ·mol^−1^ for 2,6-DCPIP, implying that anionic exchange is the mechanism that controls the adsorption process of 2,6-DCPIP [3, 57]. However, for IC (17.165 kJ·mol^−1^), this value is higher than the range. The positive values of adsorption heat evaluated from the Temkin model (Table 2) indicate the exothermic character of the adsorption process [3, 53]. Moreover, the adsorption heat of IC (14.935 kJ·mol^−1^) is greater than the adsorption heat of 2,6-DCPIP (10.712 kJ·mol^−1^). This may reflect the fact that interactions between IC ions and CTAB-modified POF are more energetic than interactions between 2,6-DCPIP ions and CTAB-modified POF [3, 58].

The separation factor values evaluated from the Langmuir model (Figure 12(c)) lie in the range 0–1 (0.036–0.270 for IC and 0.419–0.878 for 2,6-DCPIP), implying that adsorption is favorable for both dyes. This result corroborates with what was already observed with the Freundlich model. At higher initial dye concentrations, the *R*_L_ values are lower, showing that adsorption is more favorable at these concentrations for both dyes [3, 65]. The *R*_L_ values of IC are lower than those of 2,6-DCPIP, implying that the adsorption onto CTAB-modified POF is more favorable with IC rather than 2,6-DCPIP [56]. The *R*_L_ values also show that at high initial IC concentrations (0.036), the adsorption is nearly irreversible and at lower initial 2,6-DCPIP concentrations (0.878), the adsorption is nearly linear [66].

### 3.9. Adsorption Kinetic Models

#### 3.9.1. Pseudo-First-Order Model

The linear form of the pseudo-first-order model of Lagergren is expressed using the following equation [3, 16, 67, 68]:

(14) $$\begin{array}{c} \text{Log }\left(q_{\text{e}}-\;q_{\text{t}}\right)=\log\;q_{\text{e}}-\;\frac{K_{1_{\text{ads}}}}{2.303}\;t, \end{array}$$

where *q*_e_ and *q*_t_(*µ*mol · g^−1^) are the amounts of dyes adsorbed on CTAB-modified POF at equilibrium and at contact time *t*, respectively. *K*_1_ads__(min^−1^) is the rate constant of pseudo-first-order model. The slopes and intercepts of the linear plots of log(*q*_e_ −  *q*_t_) versus *t* were used to estimate the constant values of *K*_1_ads__ and *q*_e_, respectively.

#### 3.9.2. Pseudo-Second-Order Model

The linear expression of pseudo-second-order model is presented in the following equation [3, 16, 67, 68]:

(15) $$\begin{array}{c} \frac{t}{q_{\text{t}}}=\frac{1}{K_{2_{\text{ads}}}\;.\;q_{\text{e}}^{2}}+\frac{1}{q_{\text{e}}}\;t, \end{array}$$

where *K*_2_ads__(g · *µ*mol^−1^ · min^−1^) is the rate constant of the pseudo-second-order model. The slopes and intercepts of the linear plots of *t*/*q*_t_ versus *t* were used to calculate the constant values of *q*_e_ and *K*_2_ads__, respectively. From this model, the initial rate of reaction *h* (*μ*mol·g^−1^·min^−1^) (equation (16)) and the half time of the reaction *t*_1/2_ (min) (equation (17)) were evaluated:

(16) $$\begin{array}{c} h=K_{2_{\text{ads}}}.q_{\text{e}}^{2}, \end{array}$$

(17) $$\begin{array}{c} t_{1/2}=\frac{1}{q_{\text{e}}.K_{2_{\text{ads}}}}. \end{array}$$

#### 3.9.3. Elovich Model

The linear expression of the Elovich model is represented by the following equation [3, 67, 69]:

(18) $$\begin{array}{c} q_{\text{t}}=\frac{1}{\beta }\ln\left(\alpha \beta \right)+\frac{1}{\beta }\ln\;t, \end{array}$$

where *α* (*μ*mol·g^−1^·min^−1^) is the initial adsorption rate and *β* (g·*μ*mol^−1^) is related to the extent of surface coverage and activation energy for chemisorption. The slopes and intercepts of the linear plots of *q*_t_ versus ln *t* were used to determine the constant values of *β* and *α*, respectively.

#### 3.9.4. Intraparticle Diffusion Model

The intraparticle diffusion model based on the theory proposed by Weber and Morris is usually expressed in terms of square root of time, as shown in the following equation [3, 14, 25, 67]:

(19) $$\begin{array}{c} q_{t}=K_{\text{ip}}\;t^{1/2}+C, \end{array}$$

where *K*_ip_ (*μ*mol·g^−1^·min^−1/2^) and *C* (*μ*mol·g^−1^) are, respectively, the intraparticle diffusion rate constant and the thickness of the boundary layer. From the slopes and intercepts of straight lines obtained by plotting *q*_t_ versus  *t*^1/2^, the constant values of *K*_ip_ and C were estimated, respectively.

#### 3.9.5. External Mass Transfer Resistance Model

This model is used to calculate the initial rate of solute sorption using the classical mass transfer equation [3, 14, 70]:

(20) $$\begin{array}{c} \frac{\text{d}C_{\text{t}}}{\text{d}t}=-\beta_{\text{L}}S\left(C_{\text{t}}-\;C_{\text{s}}\right), \end{array}$$

where *β*_L_, *C*_t_, *C*_s_, and S are, respectively, the external mass transfer coefficient, the liquid-phase solute concentration at time *t*, the liquid-phase solute concentration at the particle surface and the specific surface area for mass transfer. The following equation is obtained by simplifying this equation [3, 14, 70]:

(21) $$\begin{array}{c} \frac{\text{d}\left(C_{\text{t}}/C_{0}\right)}{\text{d}t}\;=\;-\beta_{\text{L}}S. \end{array}$$

The initial slope of the *C*_t_/*C*_0_ versus time graph was used to approximate the external mass transfer rate *β*_L_*S*.

#### 3.9.6. Boyd Model

The Boyd model is used to evaluate the rate-controlling step involved in the dye adsorption process [3, 14, 41, 55, 70]:

(22) $$\begin{array}{c} F=1-\frac{6}{\pi^{2}}\exp\left(-\text{Bt}\right). \end{array}$$

Since  *F*=*q*_t_/*q*_e_ , Bt could be determined as follows:

(23) $$\begin{array}{c} \text{Bt}=-0.4977-\ln\left(1-F\right), \end{array}$$

where *F* and Bt are, respectively, the fraction of solute adsorbed at different times *t* and a mathematical function of *F*. The effective diffusion coefficient *D*_i_(cm^2^/s) is estimated by applying the calculated *B* values using the following relation [3, 41, 70, 71]:

(24) $$\begin{array}{c} B=\frac{\left(\pi^{2}D_{\text{i}}\right)}{r^{2}}, \end{array}$$

where *r* is the mean radius of the particle determined by sieve analysis and by assuming them as spherical particles.

**Figure 13:**
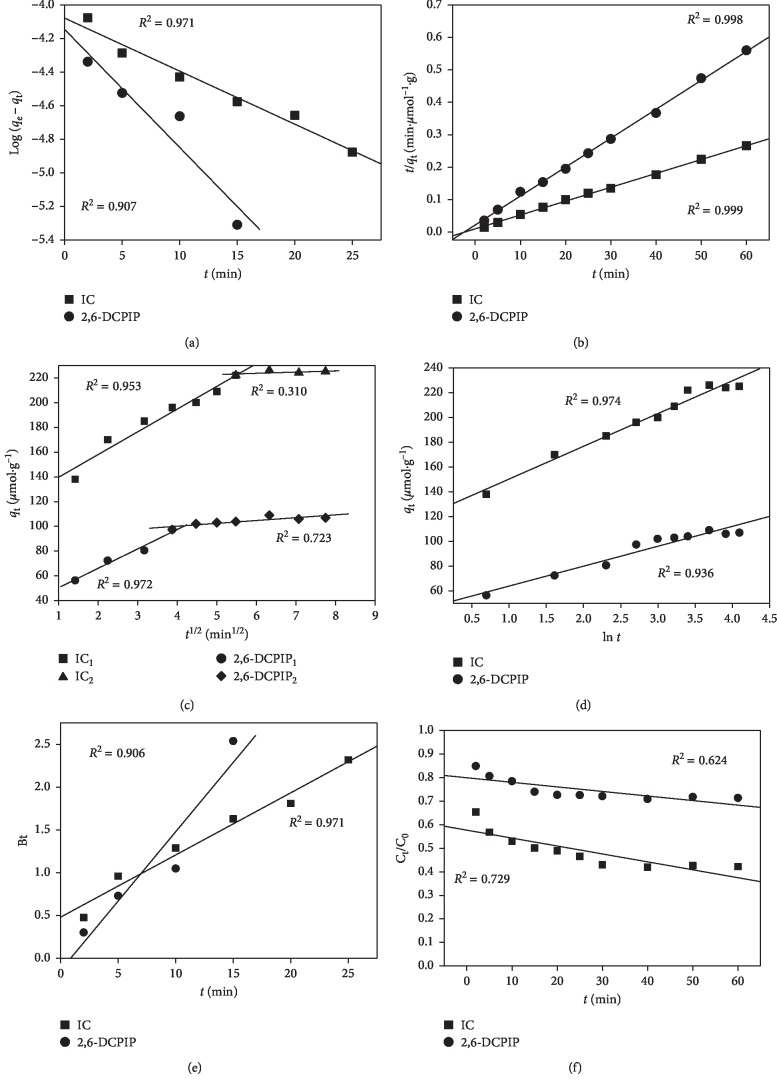
Linear regression of adsorption kinetics of (a) pseudo-first-order, (b) pseudo-second-order, (c) intraparticle diffusion, (d) Elovich, (e) Boyd, and (f) external mass transfer.

The linear regression graphs of adsorption kinetics for IC and 2,6-DCPIP adsorption onto CTAB-modified POF are shown in Figure 13, and the corresponding constants are summarized in Table 4.

The data in Table 4 indicate that, compared to the pseudo-first-order and Elovich model, the pseudo-second-order model is perfectly appropriate to describe the adsorption kinetics of IC and 2,6-DCPIP on the CTAB-modified POF with a high correlation coefficient (*R*^2^ ≥ 0.998). Moreover, it can be observed that the calculated *q*_e_ values from pseudo-second-order model are close to the experimental *q*_e_ values obtained during the adsorption process at equilibrium, indicating that the adsorption of IC and 2,6-DCPIP onto CTAB-modified POF is a process that is governed by chemisorption [1, 3]. On the other hand, the values of the initial rate of reaction and the reaction half time determined from pseudo-second-order model confirm that the adsorption onto CTAB-modified POF of IC (100.604 *μ*mol·g^−1^·min^−1^; 2.328 min) is faster than 2,6-DCPIP (45.683 *μ*mol·g^−1^·min^−1^; 2.457 min) [3, 58, 72].

As shown from Figure 13(c), the curves of intraparticle diffusion are equipped with two linear portions for both dyes, which elucidate the two adsorption stages. The first section of plots located at the beginning of the process indicates that boundary layer diffusion or film diffusion probably limited dye adsorption. In this section, the IC and 2,6-DCPIP molecules diffuse through the solution to the external surface of CTAB-modified POF. The second section of plots located at the end of the process indicates intraparticle diffusion or pore diffusion as the adsorption limiting step [3, 73]. Figure 13(c) and Table 4 also show that the first stage is faster than the second one, which may be assigned to the very slow diffusion of the IC and 2,6-DCPIP molecules from the surface film into the micropores in which the adsorption sites are less accessible. The deviation of intraparticle diffusion model curves from the origin of the graph is an indication that IC and 2,6-DCPIP molecules diffusion in the bulk of CTAB-modified POF is not the only rate-controlling step that governs the adsorption [3, 10, 17]. The values of the thickness of boundary layer C for each linear portion are different to zero, meaning that intraparticle diffusion is present in the diffusion process, but it is not the sole rate-controlling step in all the stages [3, 55]. Moreover, the larger C values at the second portion for both dyes, correspond to a greater boundary layer diffusion effect [3, 52]. In addition, a greater C value of IC (217.767 *μ*mol·g^−1^) compared to a lower C value of 2,6-DCPIP (90.936 *μ*mol·g^−1^) indicated that IC diffuses more in the interlayer of CTAB-modified POF than 2,6-DCPIP.

Figure 13(e) shows that the Boyd model straight lines deviate from the origin of the graph, signifying that external mass transfer mainly governs the rate controlling step at the initial stages [3, 14, 55, 70]. Table 4 shows that the values of *D*_i_ (3.070 × 10^−9^ cm^2^/s for IC and 6.843 × 10^−9^ cm^2^/s for 2,6-DCPIP) are found between 1 × 10^−13^ and 1 × 10^−5^ cm^2^/s, indicating that chemisorption takes place during the adsorption process [3, 72]. This result corroborates with what was already noted with pseudo-second-order and Dubinin–Radushkevich models. Thus, the adsorption of IC and 2,6-DCPIP on the CTAB-modified POF is best described by external mass transfer diffusion rather than internal diffusion. A similar observation was reported in our previous work [3].

### 3.10. Desorption

**Figure 14:**
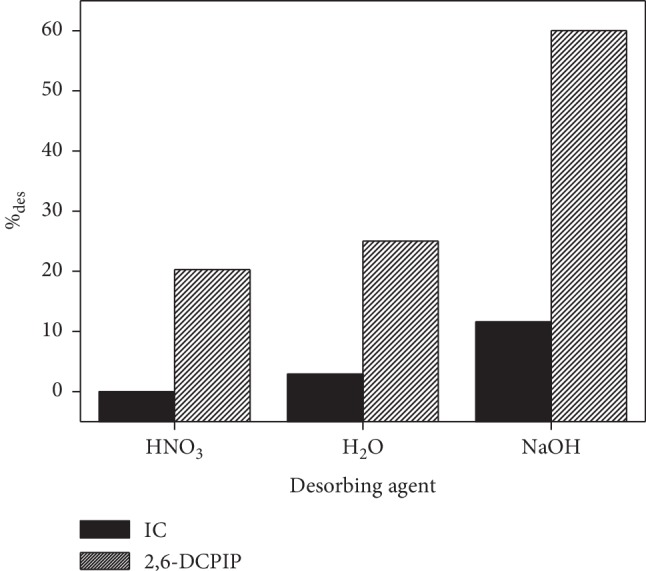
Effect of desorption solution on the recovery of IC and 2,6-DCPIP adsorbed. Experimental conditions: 4 mg of IC or 2,6-DCPIP loaded biosorbents; *G* = 0–100 *μ*m; *t* = 40 min for IC and *t* = 30 min for 2,6-DCPIP; *v* = 150 rpm; *V* = 30 mL; at room temperature.

In this study, the desorbing agents as NaOH, HNO_3_ (1 × 10^−4^ mol·L^−1^), and H_2_O were used to regenerate CTAB-modified POF. The results illustrated in Figure 14 show that the higher desorption percentage, 11.587% for IC and 60.033% for 2,6-DCPIP, is obtained in NaOH solution. This result can be explained by the phenomenon of anionic exchange between the hydroxyl ions (OH^−^) of NaOH solution and the anionic dyes loaded with CTAB-modified POF. The low desorption percentage obtained with IC compared to 2,6-DCPIP is due to the strong bond formed between the IC ions and the CTAB-modified POF [3, 26]. However, the very low desorption percentage of IC represents the fact that the IC molecules and CTAB-modified POF are bound through strong interaction leading to high stability of IC loaded with CTAB-modified POF [2, 19, 44].

## 4. Conclusions

From this study, the capacity of CTAB-modified POF for the removal of IC and 2,6-DCPIP from aqueous solution has been investigated and the following conclusions can be made. The biosorbent characterizations proved the successful impregnation of CTAB onto raw POF. The new material is an efficient biosorbent for the removal of the two dyes, but the removal of IC showed better performance than that of 2,6-DCPIP. The adsorption performances were strongly affected by various operating parameters such as treatment of biosorbent, contact time, pH of solution, initial concentration of dyes, biosorbents dosage, and ionic strength, even though the ionic strength does not affect the adsorption capacity of 2,6-DCPIP. The nonlinear regressions of adsorption isotherms were investigated, and the values of coefficient of determination and chi-square test showed that Freundlich and Temkin isotherms are most appropriate to describe the adsorption of IC and 2,6-DCPIP on CTAB-modified POF, respectively. The comparison of the adsorption of IC and 2,6-DCPIP by some adsorbents was shown, and CTAB-modified POF has a significant potential for the adsorption of dyes from an aqueous solution. Results of adsorption kinetics indicated that the adsorption processes were best described by pseudo-second-order kinetics. The diffusion mechanism was studied, and the results showed that external mass transfer was predominant in the rate controlling step. Desorption using NaOH solution as the desorbing agent recovers a maximum quantity of IC and 2,6-DCPIP and considers the reuse of CTAB-modified POF. From the results obtained, the valorization of CTAB-modified POF for the removal of anionic dyes from dye polluted wastewater such as textile effluents is promising.

## Figures and Tables

**Table 1:** Elemental analysis of raw, NaOH-treated, and CTAB-modified POF.

Biosorbents	Elemental composition (%)			
	C	H	N	O
Raw POF	42.282	6.276	0.788	50.654
NaOH-treated POF	40.532	6.161	0.263	53.044
CTAB-modified POF	49.236	8.006	1.229	41.529

**Table 2:** Langmuir, Freundlich, Dubinin–Radushkevich, and Temkin parameters for IC and 2,6-DCPIP adsorption onto CTAB-modified POF.

Dyes	Langmuir				Freundlich				Dubinin–Radushkevich				Temkin			
	*K*_L_ (L·*μ*mol^−1^)	*q*_max_ (*μ*mol·g^−1^)	*χ* ^2^	*r* ^2^	*K*_f_ (L·g^−1^)	1/*n*	*χ* ^2^	*r* ^2^	*E* (kJ·mol^−1^)	*q*_max_ (*μ*mol·g^−1^)	*χ* ^2^	*r* ^2^	Δ*q* (kJ·mol^−1^)	KT (L·*μ*mol^−1^)	*χ* ^2^	*r* ^2^
IC	0.270	275.426	14.105	0.859	0.003	0.231	4.398	0.953	17.165	736.670	4.812	0.946	14.935	6.329	5.563	0.934
2,6-DCPIP	0.014	230.423	3.462	0.967	0.046	0.629	6.291	0.938	9.880	2018.255	5.306	0.948	10.712	0.120	1.196	0.986

**Table 3:** Comparison of maximum adsorption capacities of some adsorbent materials for IC and 2,6-DCPIP, from the literature.

Adsorbent materials	Adsorbate	*q*_max_ (mg·g^−1^)	References
Palm oil fiber	IC	128.444	Present work
Pecan nut shells	IC	13.408	[59]
Calcium hydroxide	IC	0.950	[47]
Montmorillonite	IC	40.000	[60]
Fe-zeolitic tuff	IC	32.830	[61]
Carbonaceous material	IC	92.830	[61]
Chitin	IC	5.783	[62]
Chitosan	IC	71.818	[62]
Rice husk ash	IC	65.908	[46]
Layered double hydroxide	IC	55.500	[63]
Brazil nut shells	IC	1.090	[64]
Palm oil fiber	2,6-DCPIP	75.143	Present work
Palm tree trunk	2,6-DCPIP	51.299	[3]

**Table 4:** Pseudo-first order, pseudo-second-order, Elovich, external mass transfer, Boyd, and intraparticle diffusion parameters for IC and 2,6-DCPIP adsorption onto CTAB-modified POF.

Kinetic models	Constants	IC	2,6-DCPIP
Pseudo-first-order model	*q*_exp_(*µ*mol · g^−1^)	222.107	102.306
	*q*_e_(*µ*mol · g^−1^)	83.414	71.385
	*K*_1_ads__(min^−1^)	0.014	0.030
	*R* ^2^	0.971	0.907

Pseudo-second-order model	*q*_exp_(*µ*mol · g^−1^)	222.107	102.306
	*q*_e_(*µ*mol · g^−1^)	234.192	112.233
	*K*_2_ads__(g · *µ*mol^−1^ · min^−1^)	0.002	0.004
	*h* (*μ*mol·g^−1^·min^−1^)	100.604	45.683
	*t*_1/2_ (min)	2.328	2.457
	*R* ^2^	0.999	0.998

Elovich model	*α* (mmol·g^−1^·min^−1^)	2.860	0.315
	*β* (g·*μ*mol^−1^)	0.038	0.062
	*R* ^2^	0.974	0.936

External mass transfer	*β*_L_ *S* × 10^3^(min^−1^)	3.370	1.940
	*R* ^2^	0.729	0.624

Boyd model	*B*(min^−1^)	0.073	0.162
	*D*_*i*_ × 10^9^(cm^2^/s)	3.070	6.843
	*R* ^2^	0.971	0.906

Intraparticle diffusion model	*K*_ip_1__ (*μ*mol·g^−1^·min^−1/2^)	18.322	13.739
	*K*_ip_2__ (*μ*mol·g^−1^·min^−1/2^)	0.974	2.298
	*C*_1_ (*μ*mol·g^−1^)	121.445	38.601
	*C*_2_ (*μ*mol·g^−1^)	217.767	90.936
	*R*_1_^2^	0.953	0.953
	*R*_2_^2^	0.310	0.723

## Data Availability

All data included in our manuscript are available.

## References

[1] Chakraborty S., Chowdhury S., Das Saha P. (2011). Adsorption of Crystal Violet from aqueous solution onto NaOH-modified rice husk. *Carbohydrate Polymers*.

[2] Ibrahim S., Fatimah I., Ang H.-M., Wang S. (2010). Adsorption of anionic dyes in aqueous solution using chemically modified barley straw. *Water Science and Technology*.

[3] Ngaha M. C. D., Djemmoe L. G., Njanja E., Kenfack I. T. (2018). Biosorption isotherms and kinetics studies for the removal of 2,6-dichlorophenolindophenol using palm tree trunk (*Elaeis guineensis*). *Journal of Encapsulation and Adsorption Sciences*.

[4] Jalil A. A., Triwahyono S., Adam S. H. (2010). Adsorption of methyl orange from aqueous solution onto calcined Lapindo volcanic mud. *Journal of Hazardous Materials*.

[5] Hamad H. A., Sadik W. A., Abd El-latif M. M., Kashyout A. B., Feteha M. Y. (2016). Photocatalytic parameters and kinetic study for degradation of dichlorophenol-indophenol (DCPIP) dye using highly active mesoporous TiO_2_ nanoparticles. *Journal of Environmental Sciences*.

[6] Mane V. S., Vijay Babu P. V. (2013). Kinetic and equilibrium studies on the removal of Congo red from aqueous solution using Eucalyptus wood (*Eucalyptus globulus*) saw dust. *Journal of the Taiwan Institute of Chemical Engineers*.

[7] Ulker A. G., Mehtap E., Eliza T., Feride D. (2016). Mono and simultaneous removal of crystal violet and safranin dyes from aqueous solutions by HDTMA-modified Spirulina sp.. *Process Safety and Environmental Protection*.

[8] Greluk M., Hubicki Z. (2011). Comparison of the gel anion exchangers for removal of acid orange 7 from aqueous solution. *Chemical Engineering Journal*.

[9] Aki M. A., Youssef A. M., Al-Awadhi M. M. (2013). Adsorption of acid dyes onto bentonite and surfactant-modified bentonite. *Journal of Analytical and Bioanalytical Techniques*.

[10] Shanker M., Chinniagounder T. (2012). Adsorption of reactive dye using low cost adsorbent: cocoa (*Theobroma cacao*) shell. *World Journal of Applied Environmental Chemistry*.

[11] Thiyagarajan E., Saravanan P., Shiyamala Devi S. (2017). Biosorption of reactive red 2 using positively charged Metapenaeus monoceros shells. *Journal of Saudi Chemical Society*.

[12] Wan Ngah W. S., Hanafiah M. A. K. M. (2008). Removal of heavy metal ions from wastewater by chemically modified plant wastes as adsorbents: a review. *Bioresource Technology*.

[13] Yagub M. T., Sen T. K., Afroze S., Ang H. M. (2014). Dye and its removal from aqueous solution by adsorption: a review. *Advances in Colloid and Interface Science*.

[14] Benaïssa H., Elouchdi M. A. (2011). Biosorption of copper (II) ions from synthetic aqueous solutions by drying bed activated sludge. *Journal of Hazardous Materials*.

[15] Carletto R. A., Fabiana C., Francesca B., Franco F. (2008). Adsorption of Congo red dye on hazelnut shells and degradation with *phanerochaete chrysosporium*. *Bioresource*.

[16] Albadarin A. B., Mangwandi C. (2015). Mechanisms of Alizarin Red S and Methylene blue biosorption onto olive stone by-product: isotherm study in single and binary systems. *Journal of Environmental Management*.

[17] Said A. E. A., Alyl A. A. M., El-Wahab M. M. A. (2013). An efficient biosorption of direct dyes from industrial wastewaters using pretreated sugarcane bagasse. *Energy and Environmental Engineering*.

[18] Ofomaja A. E., Ho Y.-S. (2008). Effect of temperatures and pH on methyl violet biosorption by Mansonia wood sawdust. *Bioresource Technology*.

[19] Chen D., Chen J., Luan X., Ji H., Xia Z. (2011). Characterization of anion-cationic surfactants modified montmorillonite and its application for the removal of methyl orange. *Chemical Engineering Journal*.

[20] Gładysz-Płaska A., Majdan M., Pikus S., Sternik D. (2012). Simultaneous adsorption of chromium(VI) and phenol on natural red clay modified by HDTMA. *Chemical Engineering Journal*.

[21] Koswojo R., Utomo R. P., Ju Y. (2010). Acid Green 25 removal from wastewater by organo-bentonite from Pacitan. *Applied Clay Science*.

[22] Zenasni M. A., Meroufel B., Merlin A., George B. (2014). Adsorption of Congo red from aqueous solution using CTAB-kaolin from Bechar Algeria. *Journal of Surface Engineered Materials and Advanced Technology*.

[23] Dotto G. L., Buriol C., Pinto L. A. A. (2014). Diffusional mass transfer model for the adsorption of food dyes on chitosan films. *Chemical Engineering Research and Design*.

[24] Chen Z., Deng H., Chen C., Yang Y., Xu H. (2014). Biosorption of malachite green from aqueous solutions by *Pleurotus ostreatus* using Taguchi method. *Journal of Environmental Health Science and Engineering*.

[25] Chowdhury S., Saha P. (2010). Sea shell powder as a new adsorbent to remove basic green 4 (malachite green) from aqueous solutions: equilibrium, kinetic and thermodynamic studies. *Chemical Engineering Journal*.

[26] Maznah W. O. W., Al-Fawwaz A. T., Surif M. (2012). Biosorption of copper and zinc by immobilised and free algal biomass, and the effects of metal biosorption on the growth and cellular structure of *Chlorella* sp. and *Chlamydomonas* sp. isolated from rivers in Penang, Malaysia. *Journal of Environmental Sciences*.

[27] Abbas M., Kaddour S., Trari M. (2014). Kinetic and equilibrium studies of cobalt adsorption on apricot stone activated carbon. *Journal of Industrial and Engineering Chemistry*.

[28] Chizari Fard G., Mirjalili M., Najafi F. (2017). Hydroxylated *α*-Fe_2_O_3_ nanofiber: optimization of synthesis conditions, anionic dyes adsorption kinetic, isotherm and error analysis fiber: optimization of synthesis conditions, anionic dyes adsorption kinetic, isotherm and error analysis. *Journal of the Taiwan Institute of Chemical Engineers*.

[29] Güzel F., Sayğılı H., Sayğılı G. A., Koyuncu F. (2014). Decolorisation of aqueous crystal violet solution by a new nanoporous carbon: equilibrium and kinetic approach. *Journal of Industrial and Engineering Chemistry*.

[30] Nebaghe K. C., El Boundati Y., Ziat K., Naji A., Rghioui L., Saidi M. (2016). Comparison of linear and non-linear method for determination of optimum equilibrium isotherm for adsorption of copper(II) onto treated Martil sand. *Fluid Phase Equilibria*.

[31] Yargiç A. S., Sahim R. Z. Y., Ozbay N., Onal E. (2015). Assessment of toxic copper(II) biosorption from aqueous solution by chemically-treated tomato waste. *Journal of Cleaner Production*.

[32] Yazici H., Kiliç M., Solak M. (2008). Biosorption of copper(II) by *Marrubium globosum* subsp. *globosum* leaves powder: effect of chemical pretreatment. *Journal of Hazardous Materials*.

[33] Kenne Dedzo G., Péguy Nanseu-Njiki C., Ngameni E. (2012). Amperometric sensors based on sawdust film modified electrodes: application to the electroanalysis of paraquat. *Talanta*.

[34] Njine-Bememba C. B., Dedzo G. K., Nanseu-Njiki C. P., Ngameni E. (2015). Amination of pretreated ayous (*Triplochiton scleroxylon*) sawdust with two organosilanes: characterization, stability, and permselective property. *Holzforschung*.

[35] Adewuyi A., Pereira F. V. (2016). Nitrilotriacetic acid functionalized Adansonia digitata biosorbent: preparation, characterization and sorption of Pb (II) and Cu (II) pollutants from aqueous solution. *Journal of Advanced Research*.

[36] Adewuyi A., Pereira F. V. (2018). Preparation and application of EDTA-functionalized underutilized *Adansonia digitata* seed for removal of Cu(II) from aqueous solution. *Sustainable Environment Research*.

[37] Postai D. L., Demarchi C. A., Zanatta F., Melo D. C. C., Rodrigues C. A. (2016). Adsorption of rhodamine B and methylene blue dyes using waste of seeds of *Aleurites moluccana*, a low cost adsorbent. *Alexandria Engineering Journal*.

[38] Saygili H., Güzel F. (2016). High surface area mesoporous activated carbon from tomato processing solid waste by zinc chloride activation: process optimization, characterization and dyes adsorption. *Journal of Cleaner Production*.

[39] Calero M., Pérez A., Blázquez G., Ronda A., Martín-Lara M. A. (2013). Characterization of chemically modified biosorbents from olive tree pruning for the biosorption of leadfied biosorbents from olive tree pruning for the biosorption of lead. *Ecological Engineering*.

[40] Iddou A., Hadj Youcef M., Aziz A., Ouali M. S. (2011). Biosorptive removal of lead (II) ions from aqueous solutions using Cystoseira stricta biomass: study of the surface modification effect. *Journal of Saudi Chemical Society*.

[41] Ofomaja A. E., Naidoo E. B. (2011). Biosorption of copper from aqueous solution by chemically activated pine cone: a kinetic study. *Chemical Engineering Journal*.

[42] Yanhui Q., Junjiang T., Shuangfei W., Hao M. (2018). Amine-functionalized sugarcane bagasse: a renewable catalyst for efficient continuous flow knoevenagel condensation reaction at room temperature. *Molecules*.

[43] Ronda A., Martín-Lara M. A., Calero M., Blázquez G. (2013). Analysis of the kinetics of lead biosorption using native and chemically treated olive tree pruning. *Ecological Engineering*.

[44] Ibrahim S., Ang H.-M., Wang S. (2009). Removal of emulsified food and mineral oils from wastewater using surfactant modified barley straw. *Bioresource Technology*.

[45] Guler U. A., Sarioglu M. (2013). Single and binary biosorption of Cu(II), Ni(II) and methylene blue by raw and pretreated Spirogyra sp.: equilibrium and kinetic modeling. *Journal of Environmental Chemical Engineering*.

[46] Lakshmi U. R., Srivastava V. C., Mall I. D., Lataye D. H. (2009). Rice husk ash as an effective adsorbent: evaluation of adsorptive characteristics for Indigo Carmine dye. *Journal of Environmental Management*.

[47] Ramesh T. N., Kirana D. V., Ashwini A., Manasa T. R. (2017). Calcium hydroxide as low cost adsorbent for the effective removal of indigo carmine dye in water. *Journal of Saudi Chemical Society*.

[48] Saha P., Chowdhury S., Gupta S., Kumar I. (2010). Insight into adsorption equilibrium, kinetics and thermodynamics of Malachite Green onto clayey soil of Indian origin. *Chemical Engineering Journal*.

[49] Chen A. - H., Chen S. - M. (2009). Biosorption of azo dyes from aqueous solution by glutaraldehyde-crosslinked chitosans. *Journal of Hazardous Materials*.

[50] Namasivayam C., Kavitha D. (2002). Removal of Congo red from water by adsorption onto activated carbon prepared from coir pith, an agricultural solid waste. *Dyes and Pigments*.

[51] Aldegs Y., Elbarghouthi M., Elsheikh A., Walker G. (2008). Effect of solution pH, ionic strength, and temperature on adsorption behavior of reactive dyes on activated carbon. *Dyes and Pigments*.

[52] Elass K., Laachach A., Alaoui A., Azzi M. (2011). Removal of methyl violet from aqueous solution using a stevensite-rich clay from Morocco. *Applied Clay Science*.

[53] Sangeetha S., Piyush C., Juhee S. (2014). Investigation on biosorption of acidic dye from an aqueous solution by marine bacteria, Planococcus sp.. *International Journal of ChemTech Research*.

[54] Xue Y., Hou H., Zhu S. (2009). Adsorption removal of reactive dyes from aqueous solution by modified basic oxygen furnace slag: isotherm and kinetic study. *Chemical Engineering Journal*.

[55] Wu Z., Zhong H., Yuan X. (2014). Adsorptive removal of methylene blue by rhamnolipid-functionalized graphene oxide from wastewater. *Water Research*.

[56] Ho Y.-S., Chiang T.-H., Hsueh Y.-M. (2005). Removal of basic dye from aqueous solution using tree fern as a biosorbent. *Process Biochemistry*.

[57] Moussa A., Mohamed T. (2015). Kinetic, equilibrium and thermodynamic study on the removal of Congo red from aqueous solutions by adsorption onto apricot stone. *Process Safety and Environmental Protection*.

[58] Djemmoe L. G., Njanja T. E., Ngaha Deussi M. C., Tonle K. I. (2016). Assessment of copper(II) biosorption from aqueous solution by agricultural and industrial residues. *Comptes Rendus Chimie*.

[59] Aguayo-Villarreal I. A., Ramírez-Montoya L. A., Hernández-Montoya V., Bonilla-Petriciolet A., Montes-Morán M. A., Ramírez-López E. M. (2013). Sorption mechanism of anionic dyes on pecan nut shells (Carya illinoinensis) using batch and continuous systems. *Industrial Crops and Products*.

[60] Geyikçi F. (2016). Factorial design analysis for adsorption of Indigo Carmine onto Montmorillonite-Evaluation of the kinetics and equilibrium data. *Progress in Organic Coatings*.

[61] Gutiérrez-Segura E., Solache-Ríos M., Colín-Cruz A. (2009). Sorption of indigo carmine by a Fe-zeolitic tuff and carbonaceous material from pyrolyzed sewage sludge. *Journal of Hazardous Materials*.

[62] Prado A. G. S., Torres J. D., Faria E. A., Dias S. C. L. (2004). Comparative adsorption studies of indigo carmine dye on chitin and chitosan. *Journal of Colloid and Interface Science*.

[63] Ahmed M. A., brick A. A., Mohamed A. A. (2017). An efficient adsorption of indigo carmine dye from aqueous solution on mesoporous Mg/Fe layered double hydroxide nanoparticles prepared by controlled sol-gel route. *Chemosphere*.

[64] De Oliveira Brito S. M., Andrade H. M. C., Soares L. F., de Azevedo R. P. (2010). Brazil nut shells as a new biosorbent to remove methylene blue and indigo carmine from aqueous solutions. *Journal of Hazardous Materials*.

[65] Meroufel B., Benali O., Benyahia M., Benmoussa Y., Zenasni M. A. (2013). Adsorptive removal of anionic dye from aqueous solutions by Algerian kaolin: characteristics, isotherm, kinetic and thermodynamic studies. *Journal of Materials and Environmental Science*.

[66] Hameed B. H., El-Khaiary M. I. (2008). Malachite green adsorption by rattan sawdust: isotherm, kinetic and mechanism modeling. *Journal of Hazardous Materials*.

[67] Gusmão K. A. G., Gurgel L. V. A., Melo T. M. S., Gil L. F. (2012). Application of succinylated sugarcane bagasse as adsorbent to remove methylene blue and gentian violet from aqueous solutions: kinetic and equilibrium studies. *Dyes and Pigments*.

[68] Panda H., Tiadi N., Mohanty M., Mohanty C. R. (2017). Studies on adsorption behavior of an industrial waste for removal of chromium from aqueous solution. *South African Journal of Chemical Engineering*.

[69] Nethaji S., Sivasamy A. (2011). Adsorptive removal of an acid dye by lignocellulosic waste biomass activated carbon: equilibrium and kinetic studies. *Chemosphere*.

[70] Sharma N., Nandi B. K. (2013). Utilization of sugarcane bagasse, an agricultural waste to remove malachite green dye from aqueous solutions. *Journal of Materials and Environmental Science*.

[71] Bharathi K. S., Ramesh S. T. (2013). Removal of dyes using agricultural waste as low-cost adsorbents: a review. *Applied Water Science*.

[72] Nanseu-Njiki C. P., Kenne D. G., Ngameni E. (2010). Study of removal of paraquat from aqueous solution by biosorption onto Ayous (*triplochiton schleroxylon*) sawdust. *Journal of Hazardous Materials*.

[73] Fungaro D. A., Yamaura M., Carvalho T. E. M. (2011). Adsorption of anionic dyes from aqueous solution on zeolite from fly ash-iron oxide magnetic nanocomposite. *Journal of Atomic and Molecular Sciences*.

